# A novel microemulsion-based isotonic perfusate modulated by Ringer’s solution for improved microdialysis recovery of liposoluble substances

**DOI:** 10.1186/s12951-018-0418-2

**Published:** 2018-11-14

**Authors:** Yong-Tai Zhang, Zhi Wang, Li-Na Shen, Yan-Yan Li, Ze-Hui He, Qing Xia, Nian-Ping Feng

**Affiliations:** Department of Pharmaceutical Sciences, Shanghai University of Traditional Chinese Medicine, Shanghai, 201203 People’s Republic of China

**Keywords:** Microdialysis, Microemulsion, Biocompatibility, Nanocarrier, Solubilization

## Abstract

**Background:**

Microdialysis is promising technique for dynamic microbiochemical sampling from tissues. However, the application of typical aqueous perfusates to liposoluble substances is limited. In this study, a novel microemulsion (ME)-based isotonic perfusate (RS-ME) was prepared to improve the recovery of liposoluble components using microdialysis probes.

**Results:**

Based on pseudo-ternary phase diagrams and comparisons of the ME area, Kolliphor^®^ EL and Transcutol^®^ P were selected as the surfactant and co-surfactant, respectively, with a weight ratio (Km) of 2:1 and ethyl oleate as the oil phase. The ME was mixed with Ringer’s solution at a 1:6 ratio (v/v) to obtain the isotonic RS-ME. The droplet size distribution of the ME in RS-ME was 78.3 ± 9.2 nm, with a zeta potential of − 3.5 ± 0.3 mV. By microdialysis perfusion, RS-ME achieved higher recovery rates of the poorly water-soluble compounds evodiamine (EVO) and ruthenium (RUT), i.e., 58.36 ± 0.57% and 49.40 ± 0.57%, respectively, than those of 20% (v/v) PEG 400 Ringer's solution (RS-PEG) and 10% (v/v) ethanol Ringer’s solution (RS-EtOH). In vivo microdialysis experiments confirmed that RS-ME captured EVO and RUT molecules around the dialysis membrane more efficiently and exhibited less spreading than RS-PEG and RS-EtOH.

**Conclusions:**

Owing to the nanosized droplets formed by lipid components in the RS-ME and the limited dispersion out of the dialysis membrane, we obtained good biocompatibility and reliable dialysis results, without affecting the tissue microenvironment. As a novel perfusate, RS-ME provides an easy and reliable approach to the microdialysis sampling of fat-soluble components.

## Background

Microdialysis is a sampling technology for assaying biochemical substances in extracellular liquids in vivo [1]. After implanting a probe into the body, a micro-perfusion pump drives a perfusate flow through a dialysis membrane to dissolve small molecules around the target tissue via a concentration gradient between the inside and outside of probes, thereby enabling effective sampling from the living tissue [2]. Common perfusates include normal saline, phosphate buffer, Ringer’s solution, and citrate glucose anticoagulant solution; the composition of ion components, pH value, osmotic pressure, and ionic strength of the perfusate should be similar to those of the internal environment of the sampling site [3, 4]. Aqueous perfusates limit microdialysis to the detection of water-soluble components. In particular, using an aqueous perfusate, it is difficult to effectively dissolve fat-soluble components, resulting in low probe recovery, difficulty in detection, and large experimental error [5]. However, fat-soluble active ingredients, including pharmaceutical agents, account for a considerable proportion of biologically active substances. To increase the recovery of fat-soluble components, macromolecular substances, such as transporters or cyclodextrins, have been added to the perfusate, but the former is expensive with poor stability and the latter increases the viscosity of the system [6, 7]. In addition, these macromolecules may block the dialysis membrane pores during long-term dialysis, which in turn reduces the dialysis efficiency. Water-miscible organic solvents, such as short-chain alcohols, are also used in perfusates, but they may diffuse into the surrounding tissues through the dialysis membrane, changing the drug distribution around the probes and reducing the reliability of test results [8]. These solvents may also cause tissue irritation.

A microemulsion is a colloidal dispersion system in which emulsion droplets of 10–100 nm are dispersed in a mutually insoluble liquid to form a thermodynamically stable, isotropic, low-viscosity, and transparent solution [9]. An oil-in-water (O/W) microemulsion is widely used to improve the solubility of hydrophobic drugs [10]. Since the microemulsion is in a liquid state, fat-soluble molecules outside the O/W microemulsion droplets can diffuse through the oil–water interface into the oil phase core within the droplet, thus showing a strong capacity for the extraction of fat-soluble ingredients [11]. Therefore, in addition to being carriers for fat-soluble drugs, O/W microemulsions are used as solvents for the extraction various fat-soluble substances [12, 13].

Owing to the excellent properties of water-in-oil (O/W) microemulsions, they are suitable perfusates for the microdialysis detection of fat-soluble ingredients. In this study, an isotonic O/W microemulsion supplemented with Ringer’s solution was formulated as a novel perfusate, and the water-insoluble drugs evodiamine (EVO) and rutaecarpine (RUT) were used as models to evaluate stability, safety, and probe recovery. The feasibility of the Ringer’s solution-adjusted microemulsion (RS-ME) as a novel perfusate for the microdialysis sampling of fat-soluble ingredients was systematically evaluated.

## Results and discussion

### Preparation and stability of the novel microemulsion-based perfusate

The microemulsion consisted of four components: oil, water, a surfactant, and a co-surfactant [14]. Ethyl oleate with stable physicochemical properties was selected as the oil phase. Ethyl oleate is a suitable solvent for steroids and other lipophilic drugs, and its properties are similar to those of almond oil and peanut oil. However, ethyl oleate is less viscous and more easily absorbed by the body than fatty oils. It is used as a solvent for subcutaneous and intramuscular injections and as a structural component of biodegradable microcapsules for subdermal implantation and cyclosporine microemulsions [15]. Ethyl oleate has low toxicity and causes minimal tissue irritation. There are no reports of ethyl oleate-induced muscle stimulation [16]. Our previous studies have confirmed that ethyl oleate has good solubilization capacities for EVO and RUT, which is beneficial for microdialysis probe recovery [17].

**Fig. 1:**
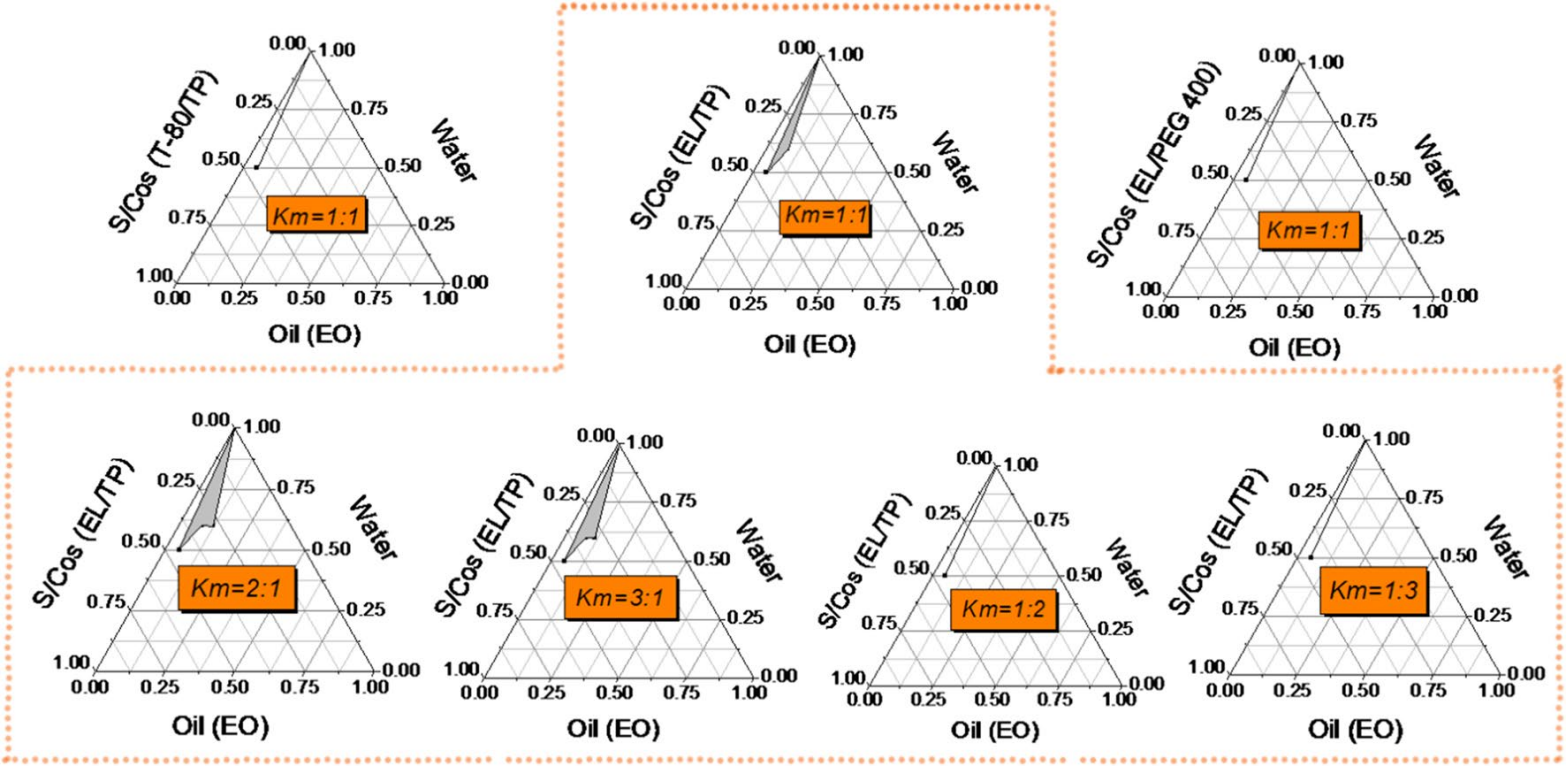
Pseudo-ternary phase diagram with Tween 80 (T-80) and Kolliphor^®^ EL (EL) as surfactants (S), Transcutol^®^ P (TP) and PEG 400 as co-surfactants (Cos), and ethyl oleate (EO) as the oil phase (Oil). Km is the mass ratio of the surfactant to co-surfactant, and the shaded area in the diagram is the oil in water (O/W) microemulsion region

To prepare the novel perfusate, Ringer’s solution was used to form an isotonic solution; the prepared microemulsion must withstand the dilution of a large volume of Ringer’s solution, without demulsification. Surfactants are critical for the formation of microemulsions. Non-ionic surfactants are less toxic than ionic surfactants, have a low critical micelle concentration and strong emulsification ability, and are not substantially affected by changes in ionic strength and pH [18]. Therefore, they are frequently used in microemulsions, followed by amphiphilic surfactants and anionic surfactants, while Tween and polyoxyethylene alkyl castor oil are commonly used in nonionic surfactants [19, 20]. In our study, Polyethylene glycol (PEG)-35 castor oil (Kolliphor^®^ EL) and Tween 80 were used as surfactants to prepare microemulsions, and Diethylene glycol monoethyl ether (Transcutol^®^ P) and polyethylene glycol (PEG) 400 were used as co-surfactants to compare the O/W microemulsion-forming regions by plotting pseudo-ternary phase diagrams. The surfactant and the co-surfactant were uniformly mixed according to a certain weight ratio (Km) to prepare a mixed surfactant. Mixtures of the oil phase (ethyl oleate) and the mixed surfactant with mass ratios of 1:9, 2:8, 3:7, 4:6, 5:5, 6:4, 7:3, 8:2, and 9:1 were prepared. Water was added dropwise with magnetic stirring. The amounts of water from clarification to turbidity and from turbidity to clarification were recorded, and a pseudo-ternary phase diagram was drawn according to the amounts of oil, water, and mixed surfactants using Origin 8.0 software. Figure 1 shows the O/W microemulsion region in the pseudo-ternary phase diagram. For the same Km value (1:1), using Kolliphor^®^ EL and Transcutol^®^ P as the surfactant and co-surfactant, respectively, the microemulsion region was significantly greater than that for Tween 80 as the surfactant and PEG 400 as the co-surfactant. Kolliphor^®^ EL has a very low critical micelle concentration of only 0.009%, while Tween 80 has a higher critical micelle concentration of 0.01%, indicating that the former has stronger emulsification performance [21]. Transcutol^®^ P has a hydrophilic–lipophilic balance (HLB) of 4.2, as a lipophilic surfactant, while PEG 400, Kolliphor^®^ EL, and Tween 80 have HLB values of 11.3, 13.5, and 15, respectively, all of which are hydrophilic surfactants [22, 23]. Although the HLB value for Transcutol^®^ P was smaller than that for PEG 400, the combination of Transcutol^®^ P and Kolliphor^®^ EL can better adjust the HLB value of the system, more effectively reducing tension and enhancing flexibility at the oil–water interface to obtain a larger microemulsion region.

Kolliphor^®^ EL and Transcutol^®^ P were selected as mixed surfactants, and ethyl oleate was used as the oil phase to prepare the microemulsion. As the Km value decreased, the microemulsion region decreased. When Km was less than 1:1, it was difficult to obtain the microemulsion; it is possible that the greater amount of Transcutol^®^ P than Kolliphor^®^ EL sharply decreased the HLB value of the system and weakened the emulsification ability, thus making it difficult to effectively reduce the oil–water interfacial tension. When the Km value was greater than 2:1, there was no significant increase in the microemulsion-forming region, indicating that excess surfactant does not significantly increase microemulsion production.

**Fig. 2:**
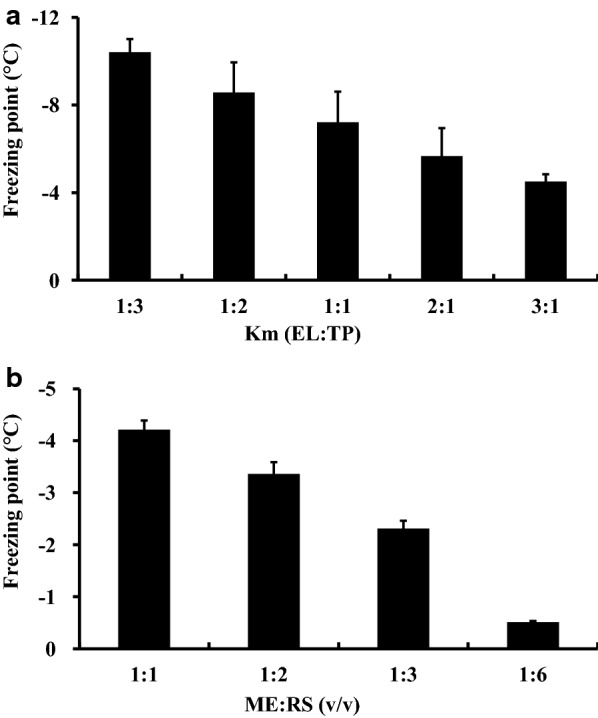
Freezing point of the microemulsion prepared using Kolliphor^®^ EL (EL) as the surfactant (S) and Transcutol^®^ P (TP) as the co-surfactant with different Km (EL/TP, w/w) values (**a**), and dilution of the microemulsion (ME) with various volumes of Ringer’s solution (RS) (**b**)

The freezing points of the microemulsions prepared with different Km values decreased as the amount of Kolliphor^®^ EL increased (Fig. 2a). To use less surfactant and to prepare a microemulsion with a lower freezing point, Kolliphor^®^ EL and Transcutol^®^ P with a Km value of 1:1 and a weight ratio of the mixed surfactant to the oil phase of 9:1 were used to prepare the novel perfusate. The freezing point of the microemulsion decreased by dilution with Ringer’s solution (Fig. 2b). When the volume ratio of the microemulsion to Ringer’s solution was 1:6, the freezing point of the system was − 0.51 ± 0.02 °C, which was close to the freezing point of plasma (− 0.52 °C).

**Fig. 3:**
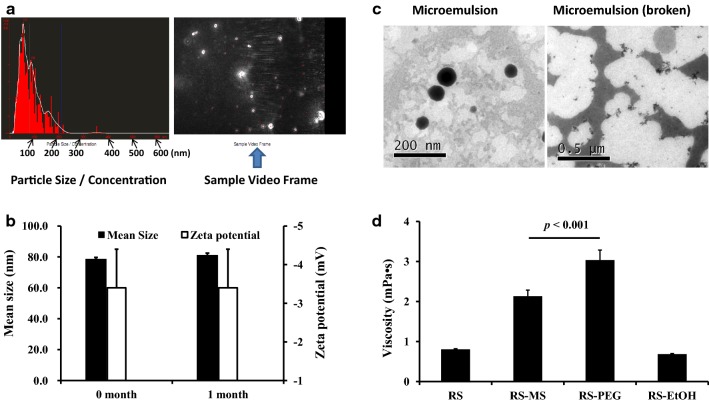
Characteristics of the Ringer’s solution-adjusted microemulsion-based isotonic perfusate (RS-ME), including droplet size distribution, as determined by NanoSight detection (**a**), the mean size and zeta potential of the RS-ME after storage for 0 and 1 month at 25 °C (**b**), transmission electron microscope imaging of the RS-ME (microemulsion) and its demulsification state after adding dimethyl sulfoxide (**c**), and the viscosity of the perfusate at 25 °C (RS, Ringer’s solution; RS-PEG, 20% [v/v] PEG 400 Ringer’s solution; RS-EtOH, 10% [v/v] ethanol Ringer’s solution) (**d**)

The newly prepared perfusate (microemulsion/Ringer’s solution = 1:6, v/v) was characterized by a mean droplet size of 78.3 ± 1.2 nm (Fig. 3a) and a zeta potential of − 3.5 ± 0.1 mV. Electrostatic repulsion delays coalescence, flocculation of the suspensions, and phase separation; this is why zeta potential is often considered an indicator of microemulsion stability. It is generally believed that the system is stable when the absolute value of zeta potential is greater than 30 mV, but zeta potential does not fully reflect the stability of the microemulsion. For microemulsion systems containing nonionic surfactants, steric hindrance plays an important role in stability. The stability of microemulsions with a low absolute value of zeta potential is still good, consistent with our findings [24]. The RS-ME was physicochemically stable after storage at 25 °C for 1 month, as evidenced by the lack of differences respectively in microemulsion droplet size distribution and zeta potential between 0 and 1 month (p > 0.05) (Fig. 3b). In addition, centrifugation indicated no delamination of the RS-ME, indicating that the novel perfusate possessed good physical stability.

The microemulsion was spherical in shape and was evenly distributed without adhesion in the TEM field; droplets were broken by the addition of DMSO (Fig. 3c). The pH value of the novel perfusate was 6.2 ± 0.6. The viscosity of RS-ME was greater than that of Ringer’s solution but significantly lower than that of 20% PEG 400 Ringer’s solution (p < 0.01) (Fig. 3d), suggesting better fluidity. The lower viscosity of RS-ME may facilitate the diffusion of analyte molecules around the dialysis membrane into to the perfusate during microdialysis.

### Microdialysis

Microdialysis is a reliable tool for monitoring endogenous and exogenous substances in the fluids of almost every tissue and organ in vivo [25]. Probe recovery is an important property of microdialysis systems [26]. Assuming that the perfusate can effectively dissolve the analyte, probe recovery mainly depends on the perfusate flow in the microdialysis probe [27]. When the perfusate flow is slow, analyte diffusion between the probe and the tissue fluid is closer to equilibrium, leading to a higher recovery rate. However, when the perfusate flow rate is too low, an insufficient amount of dialysate is collected for determination during the sampling interval. Therefore, a suitable perfusion flow should maximize probe recovery and ensure an adequate volume of dialysate for detection [28]. The flow rate is usually set to 0.1–5 μL/min. Additionally, the perfusate flow should not exceed 5–10 μL/min because the outflow of perfusate with high pressure may cause tissue damage and insufficient material exchange on both sides of the dialysis membrane.

**Fig. 4:**
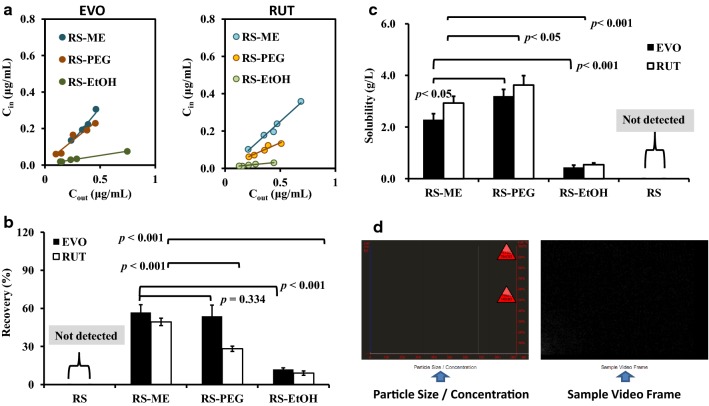
In vitro microdialysis and solubility analyses. Linear curve of the drug concentration in the dialysate (C_in_) against the drug concentration in the medium outside of the probe membrane (C_out_) (**a**), microdialysis probe recoveries of evodiamine (EVO) and rutaecarpine (RUT) using different perfusates (RS, Ringer’s solution; RS-ME, Ringer’s solution-adjusted microemulsion-based isotonic perfusate; RS-PEG, 20% [v/v] PEG 400 Ringer’s solution; RS-EtOH, 10% [v/v] ethanol Ringer’s solution) (**b**), solubility of EVO and RUT in the perfusates at 25 °C (**c**), and NanoSight tracking analysis of the medium treated with the microdialysis probe dialyzed by RS-ME for 10 h (**d**)

In a previous study, we confirmed that EVO and RUT recovery and delivery were equal at a perfusate flow of 0.2 mL/h (3.33 μL/min), and sufficient dialysate for HPLC analysis could be obtained within 30 min [17]. In this study, RS-ME, 20% PEG 400 Ringer’s solution (RS-PEG), 10% ethanol Ringer’s solution (RS-EtOH), and Ringer’s solution (RS) were used as perfusates. As shown in Fig. 4a, as the drug outside the probe increased, the drug in the corresponding perfusate increased, indicating a good linear relationship (*R*^2^ > 0.9; Table 1), suggesting that probe recovery is not highly affected by changes in the peripheral drug concentration. The highest drug concentration was captured in the RS-ME, followed by RS-PEG and RS-EtOH, while no EVO or RUT in RS could be detected by HPLC. The recovery of EVO and RUT using the RS-ME was significantly greater than that of the perfusate used for comparison (p < 0.001) (Fig. 4b). The solubilities of EVO and RUT in the RS-ME were both lower than those in the RS-PEG (p < 0.05); both of the ingredients showed the lowest solubilities in RS-EtOH (p < 0.001), except for RS (with levels below the HPLC detection limits for EVO and RUT) (Fig. 4c). The lower viscosity of RS-ME than RS-PEG allows the drug molecules to diffuse more easily across the microdialysis membrane, thus contributing to greater recovery. After dialysis for 10 h using the RS-ME as a perfusate, no nano-scale droplets were found in the medium outside of the dialysis membrane (Fig. 4d), indicating that the droplets in the RS-ME were stable and retained by the dialysis membrane to avoid diffusion outside of the probe. However, PEG400 and ethanol molecules dispersed in the RS-PEG and RS-EtOH may diffuse through the dialysis membrane, reducing the stability of the perfusate and affecting the tissue microenvironment and drug distribution surrounding the probe, further increasing the error of the experiment.

**Table 1:** Summary of regression equations for the drug concentration in the dialysate (C_in_) against the drug concentration in the medium outside of the probe membrane (C_out_)

Dialysate	Regression parameters			
	EVO	*R* ^2^	RUT	*R* ^2^
RS	C_in_ = 0	–	C_in_ = 0	–
RS-ME	C_in_ = 0.731C_out_ − 0.051	0.936	C_in_ = 0.535C_out_ − 0.016	0.981
RS-PEG	C_in_ = 0.489C_out_ + 0.010	0.942	C_in_ = 0.261C_out_ + 0.006	0.926
RS-EtOH	C_in_ = 0.093C_out_ + 0.005	0.996	C_in_ = 0.058C_out_ + 0.005	0.957

EVO, evodiamine; RUT, rutaecarpine; RS, Ringer’s solution; RS-ME, Ringer’s solution-adjusted microemulsion-based isotonic perfusate; RS-PEG, 20% (v/v) PEG 400 Ringer’s solution; RS-EtOH, 10% (v/v) ethanol Ringer’s solution

**Fig. 5:**
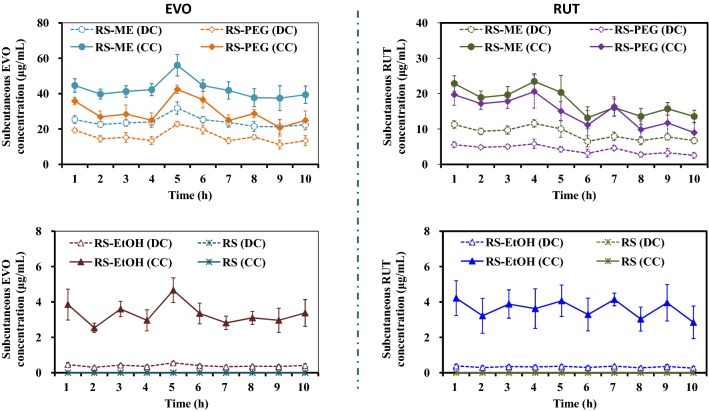
Evodiamine (EVO) and rutaecarpine (RUT) concentrations in the dialysate samples from rat subcutaneous tissues over time (DC, drug concentration by direct detection; CC, drug concentration determined by microdialysis probe recovery) based on in vivo microdialysis using various perfusates (RS, Ringer’s solution; RS-ME, Ringer’s solution adjusted microemulsion-based isotonic perfusate; RS-PEG, 20% [v/v] PEG 400 Ringer’s solution; RS-EtOH, 10% [v/v] ethanol Ringer’s solution)

The in vivo microdialysis results showed that greater EVO and RUT were obtained using the RS-ME as the perfusate than using RS-PEG and RS-EtOH, while the drug molecules could not be detected in the dialysis samples from the RS group (Fig. 5). After recovery, fluctuations in the concentrations of EVO and RUT in subcutaneous tissues were the same as those observed by direct detection. These results show that RS-ME enables superior dialysis of fat-soluble drug molecules and has good fluidity and stability, resulting in better performance than that of the other perfusates.

**Fig. 6:**
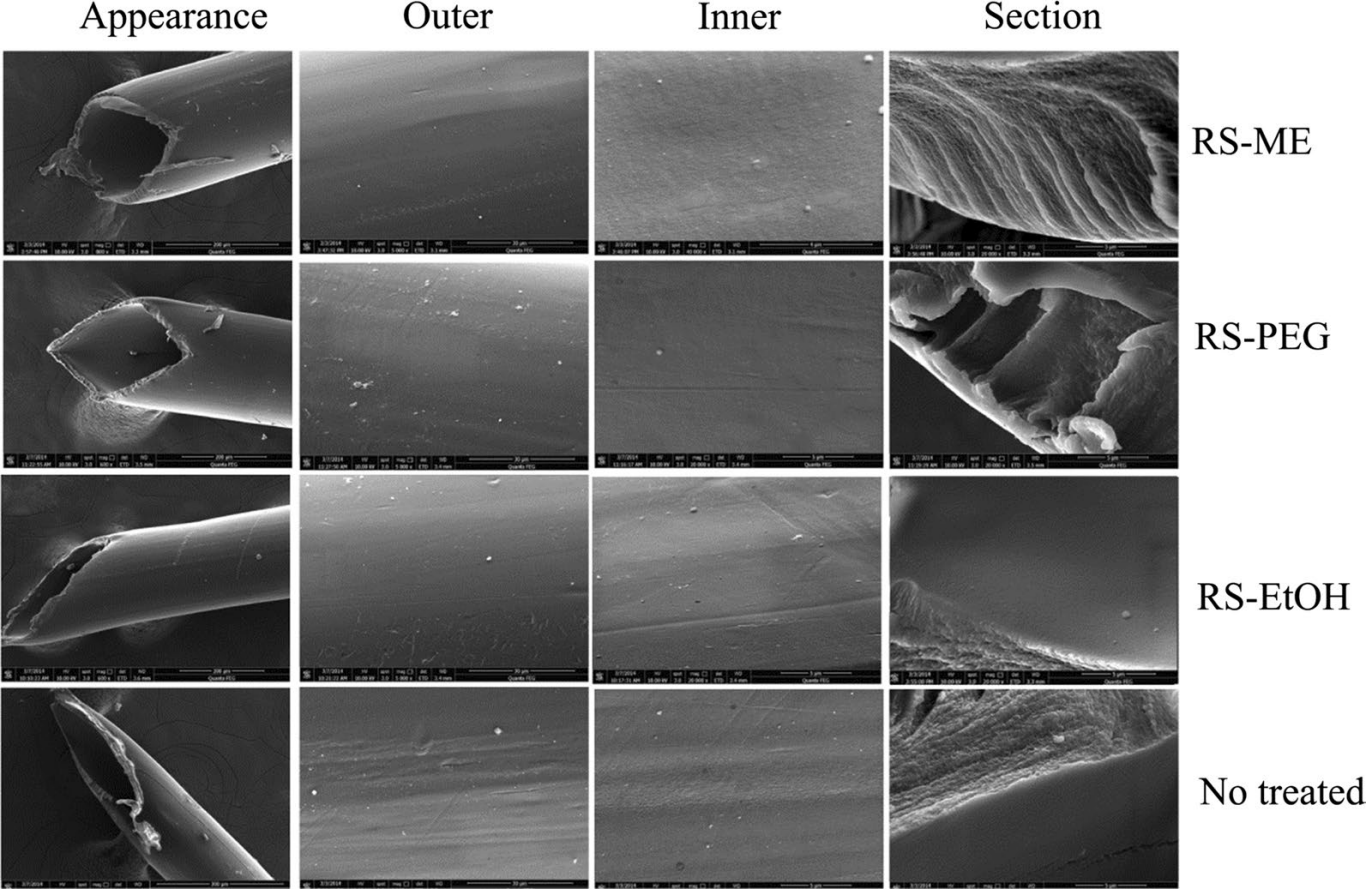
Appearance and microstructures of the microdialysis probe membranes after treatment with various perfusates (RS, Ringer’s solution; RS-ME, Ringer’s solution-adjusted microemulsion-based isotonic perfusate; RS-PEG, 20% [v/v] PEG 400 Ringer’s solution; RS-EtOH, 10% [v/v] ethanol Ringer’s solution) for 10 h

After 10 h of microdialysis, compared with the untreated dialysis membrane, the microdialysis membranes treated with RS-ME, RS-PEG, and RS-EtOH all showed better toughness; the inner and outer surfaces remained smooth and the cross sections showed no obvious structural changes (Fig. 6). The dialysis membrane tolerated the tested perfusate well, thus ensuring the reliability of the experimental results.

### Biocompatibility

**Fig. 7:**
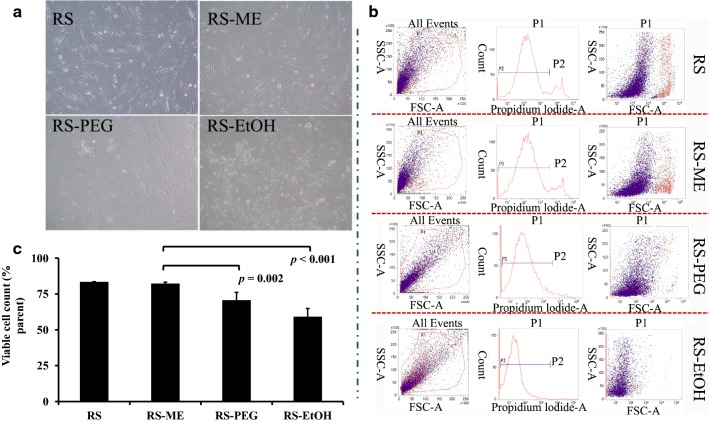
In vitro CCC-ESF-1 cell appearance (**a**), typical scatter plot of flow cytometry results for pyridine iodide-stained cells (**b**), and viable cell counts determined by flow cytometry (**c**) after incubation with various perfusates (RS, Ringer’s solution; RS-ME, Ringer’s solution-adjusted microemulsion-based isotonic perfusate; RS-PEG, 20% [v/v] PEG 400 Ringer’s solution; RS-EtOH, 10% [v/v] ethanol Ringer’s solution)

In skin microdialysis experiments, probes are usually embedded in subcutaneous tissues. Fibroblasts are the main cells in subcutaneous connective tissues. To further evaluate the safety of the perfusates, human embryonic skin fibroblasts (CCC-ESF-1) were cultured in vitro, and the perfusates were directly added to the cells for incubation. After pyridine iodide staining, dead cells emit strong red fluorescence at 660 nm, and dead cells can be distinguished from living cells by flow cytometry (FCM). After incubation for 24 h, cells showed normal morphology with few dead and suspended cells in the RS group; however, cells in the RS-ME and RS-PEG groups exhibited slight shrinkage with some cell death. The RS-EtOH group had more cell death (Fig. 7a). Figure 7b shows representative FCM scatter plots. Living cells in all groups exceeded 80%, except in the RS-EtOH group (Fig. 7c), suggesting that RS-ME and RS-PEG had good biocompatibility with cells at the tested concentrations.

**Fig. 8:**
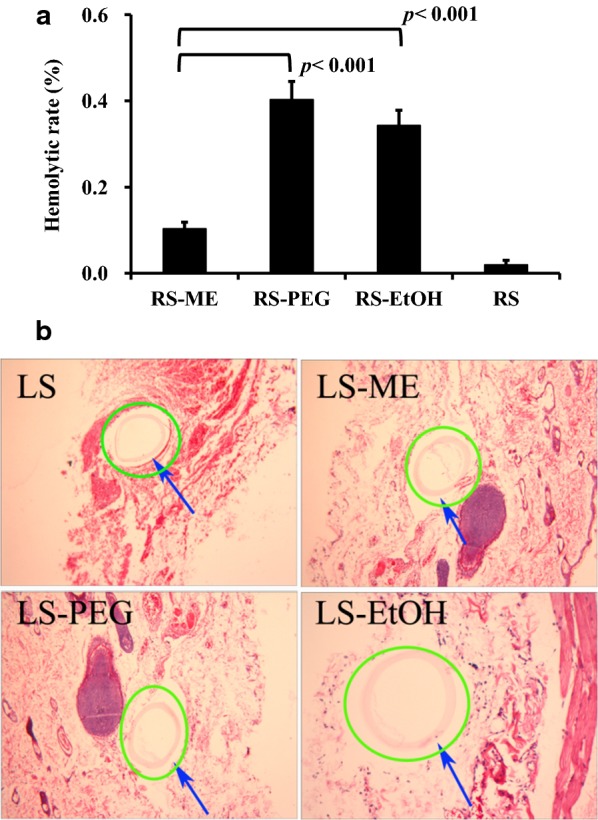
In vitro hemolytic rate for the perfusates (RS, Ringer’s solution; RS-ME, Ringer’s solution-adjusted microemulsion-based isotonic perfusate; RS-PEG, 20% [v/v] PEG 400 Ringer’s solution; RS-EtOH, 10% [v/v] ethanol Ringer’s solution) (**a**) and hematoxylin and eosin stained tissue slices containing the microdialysis probe membrane (**b**)

During microdialysis, components of the perfusate may diffuse through the dialysis membrane into the tissue. Based on a hemolytic assay, the hemolytic activity of the perfusate released through the dialysis membrane was very low and did not cause acute hemolysis according to ISO/TR7405-1984 (E) [29]. Notably, the hemolysis rate of the RS-ME group was significantly lower (p < 0.001) than those for the RS-PEG and RS-EtOH groups (Fig. 8a). RS-ME components were present in the form of droplets that do not easily penetrate the dialysis membrane, conferring better safety to the tissues around the probe.

After 10 h of microdialysis, no obvious inflammation and blood stasis were found in the skin around the dialysis membranes (Fig. 8b), indicating that the dialysis membranes had good biological safety and the perfusate had no effect on the surrounding tissues.

## Conclusions

The novel perfusate prepared by microemulsion substantially improved the microdialysis probe recovery of fat-soluble ingredients compared with existing perfusates and had good biocompatibility and physicochemical stability. It resolves the limited microdialysis recovery of poorly soluble lipid ingredients using a cutaneous perfusate. The microemulsion-based novel perfusate reduces the dependence on ultra-high sensitivity detection techniques, such as multi-stage mass spectrometry, in microdialysis experiments by substantially increasing the probe recovery of fat-soluble components, making it possible to use common detection methods, such as HPLC, thereby extending the practical applications of microdialysis technology.

## Methods

### Materials

EVO and RUT (purity 98%) were supplied by Linuo Biotechnology Co., Ltd. (Zhengzhou, China). Diethylene glycol monoethyl ether (Transcutol^®^ P) was obtained from Gattefossé (Lyon, France). Polyethylene glycol (PEG)-35 castor oil (Kolliphor^®^ EL) was a gift from BASF (Ludwigshafen, Germany). Ringer’s solution was purchased from Minsheng Pharma (Hangzhou, China). The materials used for in vitro cell studies were all obtained from Shanghai Usen Biotechnology (Shanghai, China). All other chemicals were obtained from Sinopharm Chemical Reagent Co. Ltd. (Shanghai, China) and were of high performance liquid chromatography (HPLC) or analytical grade.

### Animals and cell line

Male Sprague–Dawley rats weighing 180–220 g were used. The animal study was approved by the Animal Ethical Committee, Shanghai University of Traditional Chinese Medicine, by holding the Laboratory Animal Use License (SYXK [Hu] 2014-0008) issued by the Shanghai Science and Technology Commission.

Human embryonic skin fibroblasts (CCC-ESF-1) were obtained from the Shanghai Institute of Biochemistry and Cell Biology (Shanghai, China).

### HPLC assay

The LC-2010A HT Liquid Chromatography System (Shimadzu Corporation, Kyoto, Japan) was used to detect Evo and Rut with a C18 reverse phase column (5 µm, 4.6 mm inner diameter × 25 cm). The mobile phase was acetonitrile:water:tetrahydrofuran:acetic acid (41:59:1:0.2, v/v/v/v) with a flow rate of 1 mL/min with a detection wavelength of 225 nm. The intra-day and inter-day relative standard deviations were 1.21% and 1.57% for Evo and 1.30% and 2.24% for Rut, respectively. The microdialysis samples using the microemulsion as a perfusate were tested after adding dimethyl sulfoxide (DMSO) to break the emulsion and the perfusate not formulated with ME were assayed in a timely manner, without any handling.

### Preparation of the O/W microemulsion

The microemulsion was prepared using the water titration method at 25 °C. Briefly, the ethyl oleate, surfactant, and cosurfactant were mixed, then water was added dropwise with magnetic stirring at 300 rpm. The system may occur as different states; the microemulsion was clear, transparent, and low in viscosity, the ordinary emulsion exhibited an opaque and turbid milky white state, and a viscous, non-flowing gel state was observed after tilting.

### Preparation and characterizations of the novel perfusate

The microemulsion-based isotonic perfusate was formulated by diluting ME in Ringer’s solution. The Ringer’s solution ME-based perfusate (RS-ME) was observed using a transmission electron microscope (TEM) (Philips Tecnai 12; Philips, Amsterdam, the Netherlands) as follows. Samples were dropped onto copper nets and dried for 20 min, followed by negative staining with 2% phosphotungstic acid. Samples were allowed to dry for 10 min and observed by TEM. The freezing point of the solutions was determined using a freezing point osmometer (BS100; Yida Medical Devices Co., Ltd., Hangzhou, China). The viscosities of the formulations were measured using a DV-I + Digital Viscometer (Brookfield Engineering Laboratories Inc., Middleborough, MA, USA) with the No. 1 rotor set to 100 rpm, and the pH values were determined using a Jenway Digital pH Meter (Bibby Scientific Limited, Stone, UK) at 25 °C. The size distribution of the ME droplets in the prepared perfusate was determined using the NanoSight LM10 (Malvern Panalytical, Worcestershire, UK).

The 10% (v/v) ethanol Ringer’s solution (RS-EtOH) and 20% (v/v) polyethylene glycol (PEG) 400 Ringer’s solution (RS-PEG) were prepared for comparison.

### Stability of RS-ME

The RS-ME was stored at 25 °C for 1 month, and its physical stability was evaluated by observations of phase separation and droplet size changes, and by a centrifugation stability test at 10,000 rpm for 30 min.

### Solubility of EVO and RUT

Excess EVO and RUT were added separately to different solvents, shaken at 25 ± 1 °C for 48 h, and centrifuged at 30,000×*g* for 30 min. The supernatant was diluted with methanol, and the concentration of the drug in the saturated solution was determined by HPLC.

### Microdialysis

A linear microdialysis probe with a length of 2 cm and a hollow fiber membrane (13,000 Da molecular weight cut-off, Spectra/Por^®^; Spectrum Laboratories Inc., Rancho Dominguez, CA, USA) was used, and perfusion with a flow rate of 0.2 mL/h was conducted using a micro-infusion pump (WZ-50C6; Smiths Medical, Southington, CT, USA). Recovery validation and in vivo microdialysis sampling methods were conducted in accordance with our previous reports. Briefly, the linear probe was placed in EVO or RUT aqueous solutions with different concentrations, and dialysis was performed using the perfusates. The equilibration period was 30 min, and the dialysate was then collected for 30 min. The drug concentrations in the dialysate (C_d_) and medium (C_m_) were determined by HPLC. The recovery was calculated according to Eq. 1 [30].

1 $${\text{Recovery }}\left( {\text{\%}} \right) = ({\text{C}}_{\text{d}} /{\text{C}}_{\text{m}} ) \times 100$$

For in vivo microdialysis studies, the hair on the abdominal region of an anaesthetized rat was shaved and the probe was implanted in the skin. The donor cell was stuck to the skin above the probe, and 1 mL of the EVO or RUT ethanol solution (EVO or RUT dissolved in 70% ethanol at concentrations of 0.2 mg/mL) was applied to the skin with a contact area of 1 cm^2^. Perfusion was first started for 1 h for equilibrium, then the drug was applied. Dialysate samples were collected every 30 min for 10 h and were assayed directly by HPLC.

### Structure of the probe membrane

After the microdialysis process, part of the dialysis membrane was cut off and washed with distilled water, followed by CO_2_ critical point drying and platinum sputtering. Images were obtained by scanning electron microscopy (Quanta FEG250; FEI, Hillsboro, OR, USA).

#### In vitro cytotoxicity

CCC-ESF cells in a six-well culture dish at 5 × 10^5^ cells/well were incubated at 37 °C with 5% CO_2_ for 12 h. Then, 200 μL of medium was removed from each well and replaced with 200 μL of the tested solutions. After incubation for 24 h (37 °C, 5% CO_2_), the cells were collected, resuspended in PBS, sonicated at 20 kHz for 10 s, and treated with propidium iodide aqueous solution (1 mg/mL) for 5 min. Samples were assayed using a flow cytometer (Becton–Dickinson and Company, Canaan, CT, USA).

### Hemolysis test

Fibrin in fresh rat blood was removed, diluted with a tenfold volume of normal saline, and centrifuged at 250×*g* for 5 min. The precipitated red blood cells were collected and washed 3 times with normal saline. The red blood cells were formulated into a 2% suspension (v/v) with normal saline. One milliliter of the cell suspension was placed in an infusion tube to simulate blood vessels. The microdialysis probe was implanted into the red blood cell suspension in the infusion tube (internal diameter: 2 mm) and perfused with the prepared novel perfusate for 2 h. At the end of the experiment, the red blood cell suspension was removed and centrifuged at 250×*g* for 5 min. The supernatant was obtained after centrifugation from red blood cells and diluted in a 2% suspension with normal saline and distilled water as negative and positive controls. The absorption (A) of the supernatant collected from the novel perfusate samples (NP) and negative (NC) and positive (PC) controls was measured using an ultraviolet spectrophotometer (UV765; Shanghai Jingmi Scientific instruments Co., Ltd., Shanghai, China) at a wavelength of 540 nm. The hemolysis rate (HR) was calculated according to Eq. 2 [31].

2 $${\text{HR }}\left( {\text{\%}} \right) = \frac{{\left( {{\text{A}}_{\text{NP}} - {\text{A}}_{\text{NC}} } \right)}}{{\left( {{\text{A}}_{\text{PC}} - {\text{A}}_{\text{NC}} } \right)}} \times 100$$

### Dermal irritation

Rats were sacrificed after microdialysis was completed. The drug was removed from the skin, and the skin was excised, washed with normal saline, and fixed in 4% (w/v) polyoxymethylene for 48 h. Skins slices were embedded in paraffin and stained with hematoxylin and eosin. An optical microscope (BH-2; Olympus Corporation, Hatagaya, Japan) was used to observe skin tissues.

### Data analysis

Data are presented as mean values ± standard deviation. Significant differences were evaluated by Student’s *t*-tests, with p < 0.05 indicating significance.
